# Long-Term Effects of Goshajinkigan in Prevention of Diabetic Complications: A Randomized Open-Labeled Clinical Trial

**DOI:** 10.1155/2014/128726

**Published:** 2014-04-09

**Authors:** K. Watanabe, A. Shimada, K. Miyaki, A. Hirakata, K. Matsuoka, K. Omae, I. Takei

**Affiliations:** ^1^Center for Kampo Medicine, Faculty of Environment and Information Study, Keio University School of Medicine, 5322 Endo, Fujisawa, Kanagawa 252-0882, Japan; ^2^Division of Internal Medicine, Saiseikai Central Hospital, 1-14-17 Mita, Minato-ku, Tokyo 108-0073, Japan; ^3^Department of Clinical Research and Informatics, National Center for Global Health and Medicine, 1-21-1 Toyama, Shinjuku-ku, Tokyo 162-8655, Japan; ^4^Department of Ophthalmology, Kyorin University School of Medicine, 6-20-2 Shinkawa, Mitaka, Tokyo 181-8611, Japan; ^5^Department of Preventive Medicine and Public Health, Keio University School of Medicine, 35 Shinanomachi, Shinjuku-ku, Tokyo 160-8582, Japan; ^6^Tokyo Dental College, Ichikawa General Hospital, 5-11-13 Sugano, Ichikawa, Chiba 272-8513, Japan

## Abstract

*Objective*. This clinical trial was designed to investigate whether goshajinkigan reduces the onset of diabetic complications or not. *Materials and Methods*. A total of 332 type 2 diabetic mellitus patients were registered from 9 clinical centers from March 2000 to August 2007. Patients were randomly assigned to take goshajinkigan extract powder, 2.5 grams for 3 times a day or no kampo therapy, additionally to the regular treatment. The primary endpoints were the onset of macrovascular diseases or progression of nephropathy or retinopathy. Statistical analysis was performed by the intention-to-treat method. *Results*. After 5 years of observation, 116 patients were submitted to analysis. Among them, no macrovascular events were observed in both groups. Although 43 participants had upstaging of retinopathy or nephropathy in total, there was no significant difference between goshajinkigan group and control group. Deterioration of ankle reflex was suppressed in goshajinkigan group. Also glycated hemoglobin, and fasting plasma glucose were decreased in the goshajinkigan group. *Conclusion*. Although the power of analysis was too low to demonstrate any effects of goshajinkigan on the progression of macrovascular diseases, retinopathy or nephropathy, goshajinkigan may be beneficial for diabetic neuropathy and glycemic control.

## 1. Introduction

Diabetes mellitus has become a big problem all over the world. Also in Japan, the number of patients with diabetes mellitus has steadily increased on an annual basis. In 1998, the Ministry of Health and Welfare estimated that there were 6.9 million diabetics in Japan. Afterwards, this estimate was increased to 7.4 million and 8.9 million in 2002 and 2007, respectively [1]. According to the Diabetes Atlas (the International Diabetes Federation) published in 2011, the number of patients with diabetes living in Japan was 10.7 million [2].

Several studies have shown that the progression of diabetic microvascular complications, such as retinopathy, nephropathy, and neuropathy, can be inhibited by a good glycemic control [3–5]. However, several recent large scale randomized clinical trials have shown that glycemic control alone was not sufficient to prevent the progression of macrovascular complications, such as cerebral and myocardial infarction [5–9]. Furthermore, in terms of arteriosclerosis and microcirculation disorders, elderly individuals often have a number of risk factors for cardiovascular events, including hypertension and dyslipidemia [9]. However, protective effects of antihypertensive therapy and lipid control have been found to have limited effects for preventing macrovascular complications. Hence, an improvement in lifestyle is required, such as smoking cessation and weight control. Given the above, preventing the progression of complications only by glycemic control in the elderly is difficult, and new treatment methods are needed [10].

Clinical conditions similar to diabetes were treated with the administration of hachimijiogan in the Jingui Yaolue, a Chinese classic written approximately 1,800 years ago. Goshajinkigan, a kampo product, contains 10 herbal ingredients adding two herbs, that is, Achyranthis radix and Plantaginis semen, to hachimijiogan and is applied to patients with lumbago, edema of the lower legs, dysuria, and so forth. In an animal model of diabetes, goshajinkigan showed inhibition of weight gain, inhibitory effects on blood glucose, suppression of microalbuminuria [11], preventative effects on hypertriglyceridemia [12], inhibition of platelet aggregation [13], and elevation of the pain threshold [14, 15], all of which appeared to be effective in preventing further complications from diabetes. To date, however, no report has been published on the preventive effects of goshajinkigan for diabetic complications, with the exception of neuropathy and corneal disorders [16]. As such, a large study is needed for investigating whether kampo is effective in preventing the progression of diabetic complications.

This trial was specifically designed to determine whether a kampo formulation of goshajinkigan would reduce the rate of cardiovascular events, microvascular complications, and laboratory abnormalities, as compared with a control group without this kampo formulation in middle-aged and older people with type 2 diabetes mellitus and additional cardiovascular risk factors. Here, we report the effects of the kampo formulation on the primary composite outcome of major cardiovascular events and microvascular complications in middle-aged and older people with type 2 diabetes mellitus.

## 2. Methods

### 2.1. Eligibility and Study Design

This multicenter clinical study was conducted in 9 clinical centers in Japan. We recruited outpatients of type 2 diabetes mellitus of age between 40 and 75 years from March 2000 to August 2007. In order to investigate the progression of diabetic complications, glycated hemoglobin level of ≥6.5% was selected in the inclusion criteria. Exclusion criteria were a past macrovascular events, like cereberal stroke, myocardial infarction, angina pectoris, foot gangrene, or atherosclerotic obstruction. Other exclusion criteria were nephropathy with macroalbuminuria or with a serum creatinine levels of 1 mg/dL or more and proliferative and preproliferative retinopathy. Additional exclusion criteria which were related to goshajinkigan pattern were a body mass index of ≥30 kg/m^2^, 2 or more digestive system symptoms (e.g., gastrointestinal weakness, anorexia, nausea, and diarrhea), and 3 or more symptoms or activities indicative of sensitivity to heat, including a preference for dressing lightly, sweating upwards from the neck, a tendency to drink cold water, a flushed face, congestion of the eyeballs, and a high body temperature (not less than 36.7°C).

### 2.2. Intervention

All 116 patients were randomly assigned to the goshajinkigan group or control group. Randomization was accomplished by a study controller, and allocations were provided in sealed envelopes. Because of the long-term clinical study, we anticipated that the dropout rate is high in goshajinkigan group and allocation to goshajinkigan group was planned to be double of control group. The trial was open once patients were randomized. Only the readers of the fundus photographs were blinded. No placebo treatments were given.

As a background treatment, all participants received nutrition and physical activity counseling every year upon entering the study. Nutrition counseling included a recommendation that the caloric intake should not exceed the number of calories derived from multiplying the estimated body weight (kg) by a factor of 25. Current smokers and drinkers received smoking and drinking cessation counseling. All participants received glucose-lowering therapy, the same as the one before entering the study, as well as lipid-lowering therapy and/or blood pressure-lowering therapy. No restrictions were made regarding the choice of medications, with the exception that no kampo formula other than goshajinkigan could be used. The targeted glycated hemoglobin was lower than 6.9% (National Glycohemoglobin Standardization Program); targeted blood pressure was lower than 130/85; targeted body mass index was lower than 22.0 kg/m^2^; targeted total cholesterol was lower than 220 mg/dL; targeted high-density lipoprotein was higher than 40 mg/dL, according to the Japanese Diabetes Society Guide of 1999 for the treatment of diabetes, the Japanese Society of Hypertension Guidelines of 2000 for the management of hypertension, and the Japan Atherosclerosis Society Guidelines of 1997 for the diagnosis and treatment of hyperlipidemia.

Patients received instructional materials and behavioral counseling regarding diabetes care and were provided with goshajinkigan. Therapeutic regimens were individualized at the discretion of the investigators and patients on the basis of study-group assignments and response to therapy. The adverse effects of therapy were carefully audited both locally and centrally to ensure the safety of the patients.

### 2.3. Goshajinkigan

Patients in the goshajinkigan treatment group received 2.5 g of goshajinkigan extract (TJ-107; Tsumura Co., Tokyo, Japan) preprandially which included 4.5 g of the compound extracts of 10 herbal medicines:* Rehmanniae radix* (5g),* Achyranthis radix* (3g), Corni fructus (3g),* Dioscoreae rhizoma* (3g), Hoelen (3g),* Plantaginis semen* (3g),* Alismatis rhizoma* (3g),* Moutan cortex* (3g),* Cinnamomi cortex* (1g) and heat-processed* Aconiti* radix (1g). The extract product Goshajinkigan (TJ-107, Tsumura goshajinkigan extract granules) is a standardized spray-dried water extract, which includes magnesium stearate, lactose, and fructose fatty acid esters as diluents. The manufacturing process meets all requirements of the Japanese and international GMP guidelines.

### 2.4. Primary and Secondary Outcomes

The prespecified primary endpoints were the first occurrence of nonfatal myocardial infarction or nonfatal stroke or worsening of diabetic nephropathy (DN) or retinopathy (DR). The progression of DN was evaluated by the new onset of renal failure by dialysis or an increase in the amount of urinary protein. The urinary protein was calculated by dividing the morning urinary albumin by the urinary creatinine not related to any acute intercurrent illness. The amount of urinary protein was divided into 3 stages: <30 mg/gCr, 30–300 mg/gCr, and >300 mg/gCr.

The progression of DR was evaluated by standardized eye examinations conducted by ophthalmologists or optometrists, along with fundus photography of 4 standard stereoscopic fields at baseline and every year. The photographs were gathered at the Fundus Photograph Reading Center, located at the University of Kyorin (Mitaka, Tokyo), and were graded by trained personnel masked to the treatment assignments of the participants. The presence of capillary aneurysms, retinal bleeding, hard exudate, ring-like hard exudate, soft exudate, intraretinal microvascular abnormalities, venous abnormalities, and new blood vessels was evaluated. The DR stage was divided into 8 steps from normal to proliferative (3 classifications each pertained to the simple and preproliferative steps).

The study investigators also measured the effect of the intervention on body weight, blood pressure, fasting blood glucose, glycated hemoglobin, serum insulin (c-peptide reactivity in the insulin user), total cholesterol, triglyceride, high-density lipoprotein, serum creatinine, urea nitrogen, and DN as secondary outcomes. DN was evaluated by using two-way analysis of variance. The grade of the ankle reflex was classified into 3 steps: normal, decreased, and absent. Moreover, the results of the questionnaire on 10 subjective symptoms were evaluated by 4 steps, and 4 of these 10 symptoms were also evaluated with a visual analogue scale (graded from 0 to 100). The questionnaire included lightheadedness, abnormal sweating, occurrence of constipation and diarrhea, impotence, abnormal feeling like pater stuck to the soles, uncomfortable heat sensation of hands and feet, pain of hands and feet, abnormal sensation of hands and feet, cold sensation of hands and feet, and muscle cramp.

### 2.5. Safety Analyses

For monitoring side effects, aspartate aminotransferase, alanine aminotransferase, serum creatinine, urea nitrogen, uric acid, urinary blood, glucose, and ketone were investigated every 6 months. When critical side effects were suspected because of goshajinkigan, the treatment was stopped immediately, and suitable treatment was performed. Concomitantly, a report was promptly made to the research center.

### 2.6. Sample Size

We aimed to recruit 1000 cases for the goshajinkigan group and 500 for the control group. The sample size was calculated for an event rate of 6% per year, an effect of 20%, an alpha error of 5%, and a power level of 80%.

### 2.7. Statistical Analyses

All statistical analyses were conducted at the coordinating center with the use of SAS software, version 9.1 (SAS Institute). Baseline characteristics were compared in the 2 study groups with the use of chi-square tests and two-sample *t*-tests. At each assessment visit, glycated hemoglobin levels and fasting plasma glucose levels were summarized with the use of medians and interquartile ranges. Exposure to glucose-lowering drugs was summarized according to the study group as the number of patients who received a prescription for a medication. Analysis of the primary outcome was performed with the use of the log-rank method according to the intention-to-treat principle, and the occurrences of this outcome in the 2 study groups were compared with the use of hazard ratios and 95% confidence intervals. Kaplan-Meier estimates were used to obtain the proportion of patients who had an event during follow-up. Analyses of the secondary outcomes were performed with the use of log-rank methods, two-sample Wilcoxon's test, and Fisher's exact test. We report here all nominal *P* values, unadjusted for the multiplicity associated with the various tests performed for this study or the monitoring of the primary and mortality endpoints by the data and safety monitoring committee.

## 3. Results

### 3.1. Participants Registration, Allocation, Follow-Up, and Analysis

**Figure 1:**
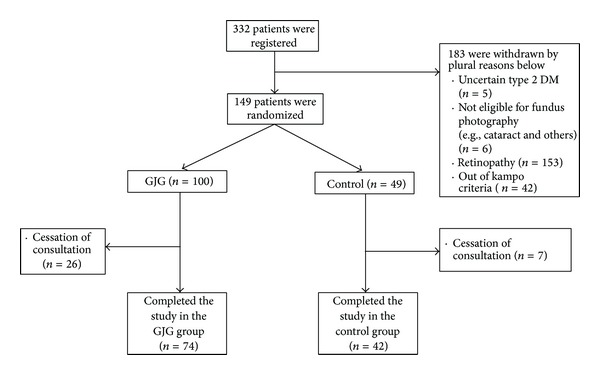
Enrollment, randomization, and follow-up of study participants. A total of 116 men and women were randomly assigned to either the goshajinkigan group or the control group. The trial was stopped when the national funding stopped, although significantly more volunteers were to be included in the trial.

A total of 332 patients were registered for this clinical trial (Figure 1), among which 162 patients were excluded because they were out of criteria. The main reason of the exclusion was the retinopathy. Even though, in the regular ophthalmological check, these patients matched the inclusion criteria, fundus photography revealed that these patients did not match the inclusion criteria. Another reason was out of goshajinkigan pattern. This was relatively difficult for the physician who contributed to this study. As a result, 170 patients were allocated to goshajinkigan (GJG) group (*n* = 100) to control group (*n* = 49).

### 3.2. Baseline Characteristics of Study Groups

A total of 116 patients were submitted for analysis of this study.  Table 1 shows the baseline characteristics of two groups. Age, sex distribution, and duration of diabetes were similar in the 2 study groups. Smoking habits were also similar. Blood chemistry profile was also similar in the 2 groups. The mean duration of follow-up was 28 months for the goshajinkigan group and 15 months for the control group.

### 3.3. Primary Outcomes

**Figure 2:**
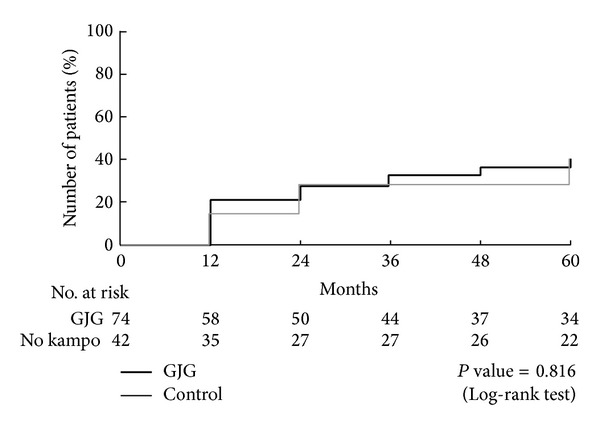
Primary endpoint. A total of 43 participants had a microvascular event and no macrovascular events: 40.2% in the goshajinkigan group and 39.1% in the control group (*P* = 0.816). The mean duration of follow-up was 28 months for the goshajinkigan group and 15 months for the control.

**Figure 3:**
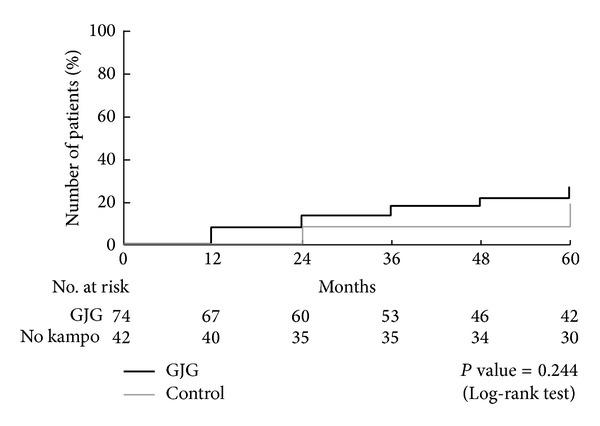
Progression of nephropathy. A total of 25 participants had nephropathy at the end of this study: 27.2% in goshajinkigan group and 18.7% in control group (*P* = 0.244).

**Figure 4:**
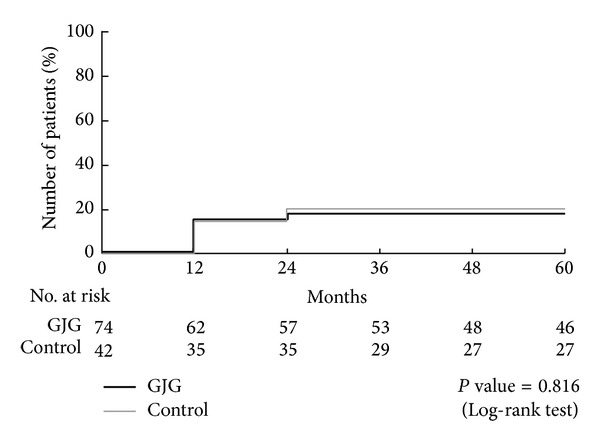
Progression of retinopathy. A total of 25 participants had retinopathy at the end of this study: 17.9% in goshajinkigan group and 20.0% in control group (*P* = 0.816).

No macrovascular events, like myocardial infarction, angina pectoris, or cerebrovascular diseases, occurred in either group. None of the patients had macrovascular events. A total of 43 participants had a progression of stages in either nephropathy or retinopathy: 40.2% in the goshajinkigan group and 39.1% in the control group (Figure 2). In terms of nephropathy, 25 participants had nephropathy at the end of this study: 27.2% in goshajinkigan group and 18.7% in control group (*P* = 0.244) (Figure 3). In terms of retinopathy, a total of 25 participants had nephropathy at the end of this study: 17.9% in goshajinkigan group and 20.0% in control group (*P* = 0.816) (Figure 4).

### 3.4. Secondary Outcomes

**Figure 5:**
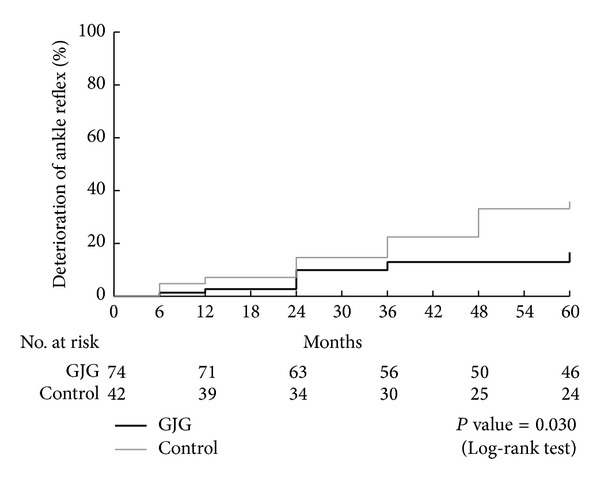
Progression of neuropathy (%). Progression of the ankle reflex was significantly decreased in the goshajinkigan group compared with the control group. The hazard ratio was calculated as 0.436 (95% CI: 0.198–0.962), and the *P* value was 0.030 as determined by the log-rank test.

The progression of the grade of the ankle reflex was significantly more frequent in the control group. The hazard ratio was calculated as 0.436 (95% CI: 0.198–0.962), and the *P* value was 0.040 as determined by the log-rank test (Figure 5).

**Figure 6:**
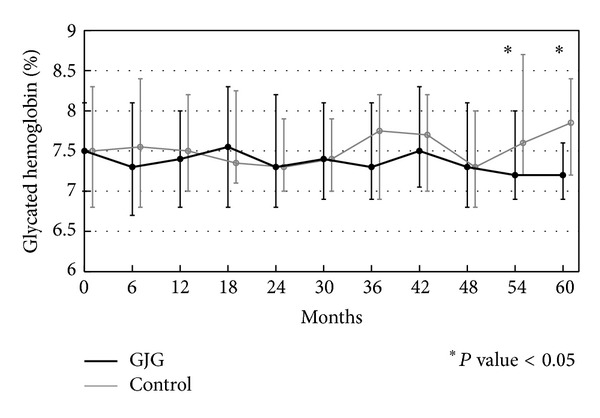
Glycated hemoglobin (%). Glycated hemoglobin was significantly decreased in the goshajinkigan group compared with the control group (no kampo treatment) at the 54th and 60th months.

**Figure 7:**
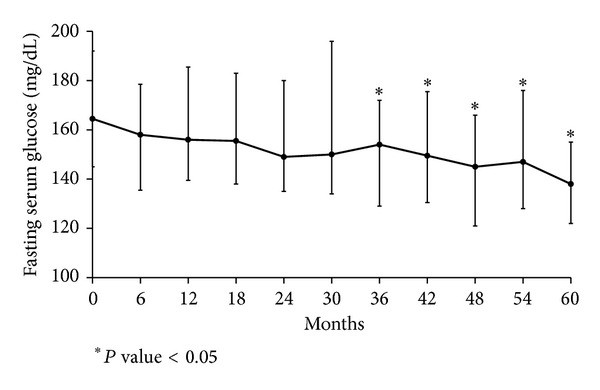
Fasting serum glucose (mg/dL). Fasting serum glucose was significantly decreased in the goshajinkigan group compared with the baseline value at the 36th, 42nd, 48th, 54th, and 60th months.

Stable median levels of glycated hemoglobin of 7.8% (interquartile range, 6.4–11.5) and 7.9% (interquartile range, 6.7–13.5) were maintained in the goshajinkigan and control groups, respectively, throughout the follow-up period. According to an exploratory examination, the glycated hemoglobin of the goshajinkigan group decreased significantly in the 60th month as compared to the control group (Figure 6). Fasting plasma glucose also decreased significantly beginning at the 36th month relative to baseline (Figure 7), while no significant changes were observed in the control group. No significant differences were observed in terms of insulin doses or oral antidiabetic medications.

Most patients had no subjective symptoms through the trial period. An analysis of only the patients who had a change in subjective symptoms was performed, in addition to an analysis of the entire set of patients. For the patients who experienced a change in symptoms, the occurrence of constipation and diarrhea was significantly improved in the goshajinkigan group. For the entire set of patients, no significant changes occurred for any of the issues addressed in the questionnaire. There was no dropout because of the side effects of goshajinkigan.

Other outcomes, including body mass index and laboratory test results, showed no significant differences.

## 4. Discussion

Goshajinkigan extract (TJ-107) is a pharmaceutical drug covered by national health insurance program. The indications include pain in low extremities, back pain, numbness, blurred vision, dysuria, pollakiuria, and edema. It is mainly used for the senile problems. Goshajinkigan is a medicine for kidney function deficiency in terms of traditional medicine. Kidney function deficiency means a loss in congenital energy. With aging, this function is deteriorated. In this case, kidney function is not the organ kidney function. Kidney keeps one's congenital energy and, with aging, this function is deteriorated. Goshajinkigan is the derivative of hachimijiogan and two additional herbs, Achyranthis Radix and Plantaginis Semen, are added.

There are studies to show either hachimijiogan or goshajinkigan is effective for diabetic complications. Yokozawa et al. investigated that hachimijiogan had a protective effect against the diabetic nephropathy in the animal models [17–19]. They speculated that the mechanism of hachimijiogan is the protection of the formation of advanced glycation end-product by Corni Fructus which is one of the ingredients of hachimijiogan [20] or suppression of oxidative stress [21].

For retinopathy, Cameron-Schaefer et al. showed the protective effect of goshajinkigan against the diabetic retinopathy [22]. They showed that lipid peroxidation was enhanced by streptozotocin-induced hyperglycemia and that goshajinkigan prevented lipid peroxidation. They showed that soluble guanylate cyclase activation is a key mechanism to decrease the lipid peroxidation.

Although it has not been shown that goshajinkigan prevented diabetic macroangiopathy, Suzuki et al. evaluated the effects of goshajinkigan on platelet aggregation in streptozotocin-induced diabetic rats. They concluded that goshajinkigan could improve platelet aggregation in diabetes through increased production of nitric oxide via bradykinin B2-receptors and muscarinic acetylcholine receptors [13].

This clinical trial was designed based on these publications. However, we found a difference between animal models and complex clinical trials. The chosen endpoint, macroangiopathy, might have been inappropriate for clinical setting in such a short period. Whilst changes of microangiopathy might not be visible anatomically on fundus photography, goshajinkigan might influence the metabolism in the retina and thus have effects on a functional level—an effect which cannot easily be studied in humans in vivo. There had been no cases of macrovascular events. And there were no differences in the progression of retinopathy and nephropathy. Because the progression of retinopathy and nephropathy takes time, we enrolled the elder type 2 diabetes patients with moderate hyperglycemia. However, 5 years follow-up time is long for patients and the final number for the analysis was only 116 cases. The power was very low compared to the original calculation. So we could not conclude any definite effects of goshajinkigan on preventing macrovascular events such as myocardial infarction or stroke, nor could we state effects on diabetic nephropathy. Recently, some surrogate markers of macroangiopathy were proposed, like flow mediated dilation, ankle brachial pressure index, or pulse wave velocity. These are covered by the Japanese national health insurance and could be in consideration as surrogate markers.

On the other hand, our results support a beneficial effect of goshajinkigan on diabetic neuropathy, which we chose as secondary endpoint. There have been a lot of manuscripts showing that goshajinkigan had a beneficial effect on diabetic neuropathy in the animal model [21] or in the clinical settings [23, 24].

The mechanism of action of goshajinkigan on diabetic neuropathy was speculated partially by the inhibition of the aldose reductase [25, 26].

Moreover, recently, goshajinkigan has been reported to relieve the peripheral neuropathy due to oxaliplatin in patients with advanced or recurrent colorectal cancer that were receiving FOLFOX therapy [27]. The author speculated that the mechanism of goshajinkigan was the promotion of the release of dynorphin and nitric oxide production. This may be explained by the varying modes of action of the different ingredient of goshajinkigan.

Our data has also shown that goshajinkigan has some beneficial effect on serum glucose and glycated hemoglobin. When we have compared the medications in two groups, the patients in the control group had more progressed medications in 5 years (nonmedication to medication or medication to insulin). Body mass index was similar at the beginning of this study, but in 5 years, GJG group kept BMI and the control group lost weight. These two facts support that GJG reduces the serum glucose compared to the control group. Because the difference of the blood serum glucose or glycated hemoglobin was observed only in the late years, we assumed that GJG itself does not affect the insulin secretion itself. An improvement of insulin resistance may be involved to decrease the blood glucose level. This speculation was supported by the previous studies [28, 29].

In large RCTs such as the Action to Control Cardiovascular Risk in Diabetes trial and the Action in Diabetes and Vascular Disease: Preterax and Diamicron MR Controlled Evaluation trial [30, 31], hypoglycemia has been noted as one of the reasons that strict glycemic control showed no benefit in lowering the incidence and mortality of macroangiopathy. If goshajinkigan can control blood glucose without any risk of hypoglycemia, patients can receive it safely over the long term. Even though the primary endpoints were not met, our data support a positive effect of goshajinkigan on diabetic neuropathy. It furthermore might positively affect glucose metabolism in the long term. Further studies are necessary with the focus on diabetic microangiopathy and especially neuropathy.

## Figures and Tables

**Table 1:** Baseline characteristics of study groups.

	Goshajinkigan group (*n* = 74)	Control group (*n* = 42)
Age (y)	59.4 ± 7.8	60.9 ± 7.4
Female sex (%)	41.9	40.0
Duration of diabetes (y)	12 ± 6.8	10.9 ± 6.1
Weight (kg)	60.2 ± 12.2	57.2 ± 11
Body mass index	23 ± 4.1	22.1 ± 3.1
Cigarette-smoking status (%)		
Current	31.1	31.0
Former	17.6	14.3
Never	48.6	50.0
Unknown	2.7	4.8
Complications (%)		
None	45.9	38.0
Hypertension	23.0	21.4
Dyslipidemia	21.6	21.4
Atrial fibrillation	2.7	2.4
Other	31.1	38.1
Unknown	1.4	2.4
Blood pressure (mmHg)		
Systolic	131.6 ± 16.1	132.1 ± 19.4
Diastolic	78.5 ± 11.5	76.8 ± 10.2
Glycated hemoglobin (%)		
Mean	7.7 ± 1.0	7.7 ± 1.1
Fasting serum glucose (mg/dL)	170.5 ± 45.3	164.7 ± 38.5
Cholesterol (mg/dL)		
Total	205.9 ± 32.8	208.4 ± 36.7
High-density lipoprotein	54.7 ± 12.0	55.2 ± 12.6
Serum triglyceride (mg/dL)	124.9 ± 88.0	103.6 ± 42.3
Medications (%)		
Insulin	17.6	16.7
Metformin	18.9	21.4
Any sulfonylurea	58.1	52.4
Any other oral hypoglycemic agents	39.2	28.6
