# Corrigendum to “Long-Term Effects of Goshajinkigan in Prevention of Diabetic Complications: A Randomized Open-Labeled Clinical Trial”

**DOI:** 10.1155/2016/9567408

**Published:** 2016-07-19

**Authors:** K. Watanabe, A. Shimada, K. Miyaki, A. Hirakata, K. Matsuoka, K. Omae, I. Takei

**Affiliations:** ^1^Center for Kampo Medicine, Faculty of Environment and Information Study, Keio University School of Medicine, 5322 Endo, Fujisawa, Kanagawa 252-0882, Japan; ^2^Division of Internal Medicine, Saiseikai Central Hospital, 1-14-17 Mita, Minato-ku, Tokyo 108-0073, Japan; ^3^Department of Clinical Research and Informatics, National Center for Global Health and Medicine, 1-21-1 Toyama, Shinjuku-ku, Tokyo 162-8655, Japan; ^4^Department of Ophthalmology, Kyorin University School of Medicine, 6-20-2 Shinkawa, Mitaka, Tokyo 181-8611, Japan; ^5^Department of Preventive Medicine and Public Health, Keio University School of Medicine, 35 Shinanomachi, Shinjuku-ku, Tokyo 160-8582, Japan; ^6^Tokyo Dental College, Ichikawa General Hospital, 5-11-13 Sugano, Ichikawa, Chiba 272-8513, Japan

In the article titled “Long-Term Effects of Goshajinkigan in Prevention of Diabetic Complications: A Randomized Open-Labeled Clinical Trial” [1], we have noticed inadvertent errors. We would like to correct the errors here. These errors do not change the scientific conclusions of the paper.

(1) The abbreviation “DN” in the third paragraph of Section 2.4 “Primary and Secondary Outcomes” did not mean “diabetic nephropathy” and should be corrected as “diabetic neuropathy.”

(2) Section 3.1 “Participants Registration, Allocation, Follow-Up, and Analysis” should be corrected as follows: A total of 332 patients were registered for this clinical trial (Figure 1), among which 183 patients were excluded because of being out of criteria. Main reason of the exclusion was the retinopathy. Even though, in the regular ophthalmological check, these patients matched the inclusion criteria, fundus photography revealed that these patients did not match the inclusion criteria. Another reason was out of goshajinkigan pattern. This was relatively difficult for the physician who contributed to this study. As a result, 149 patients were allocated to goshajinkigan (GJG) group (*n* = 100) and to control group (*n* = 49).

(3) Section 3.4 “Secondary Outcomes” should be corrected as follows: The hazard ratio was calculated as 0.436 (95% CI: 0.198–0.962), and the *P* value was 0.030 determined by the log-rank test (Figure 5).
